# Expression of alternative NADH dehydrogenases (NDH‐2) in the phytopathogenic fungus *Ustilago maydis*

**DOI:** 10.1002/2211-5463.12475

**Published:** 2018-07-05

**Authors:** Deyamira Matuz‐Mares, Genaro Matus‐Ortega, Christian Cárdenas‐Monroy, Lucero Romero‐Aguilar, Juan Carlos Villalobos‐Rocha, Héctor Vázquez‐Meza, Guadalupe Guerra‐Sánchez, Antonio Peña‐Díaz, Juan Pablo Pardo

**Affiliations:** ^1^ Departamento de Bioquímica Facultad de Medicina Universidad Nacional Autónoma de México Ciudad de México México; ^2^ Departamento de Genética Molecular Instituto de Fisiología Celular Universidad Nacional Autónoma de México Ciudad de México México; ^3^ Bioquímica de hongos Escuela Nacional de Ciencias Biológicas Instituto Politécnico Nacional Ciudad de México México

**Keywords:** alternative NAD(P)H dehydrogenases, corn smut fungi, electron transport chain, gene expression, NADH/NADPH quinone oxidoreductase, *Ustilago maydis*

## Abstract

Type 2 alternative NADH dehydrogenases (NDH‐2) participate indirectly in the generation of the electrochemical proton gradient by transferring electrons from NADH and NADPH into the ubiquinone pool. Due to their structural simplicity, alternative NADH dehydrogenases have been proposed as useful tools for gene therapy of cells with defects in the respiratory complex I. In this work, we report the presence of three open reading frames, which correspond to NDH‐2 genes in the genome of *Ustilago maydis*. These three genes were constitutively transcribed in cells cultured in YPD and minimal medium with glucose, ethanol, or lactate as carbon sources. Proteomic analysis showed that only two of the three NDH‐2 were associated with isolated mitochondria in all culture media. Oxygen consumption by permeabilized cells using NADH or NADPH was different for each condition, opening the possibility of posttranslational regulation. We confirmed the presence of both external and internal NADH dehydrogenases, as well as an external NADPH dehydrogenase insensitive to calcium. Higher oxygen consumption rates were observed during the exponential growth phase, suggesting that the activity of NADH and NADPH dehydrogenases is coupled to the dynamics of cell growth.

**Abbreviations**
6PGD: 6 phosphogluconate dehydrogenase
Aox: alternative oxidase
EtOH‐DH: ethanol dehydrogenase
EtOH‐mm: ethanol minimal medium
Fum: fumarate
G3P‐DH: glycerol 3‐phosphate dehydrogenase
G6P‐DH: glucose 6‐phosphate dehydrogenase
Glc‐mm: glucose minimal medium
HK: hexokinase
HK: hexokinase
Lac‐DH: lactate dehydrogenase
Lac‐mm: lactate minimal medium
Mal: malate
NDH‐2: alternative NADH dehydrogenases
OAA: oxaloacetate
PFK‐1: phosphofructokinase 1
PK: pyruvate kinase
Pyr: pyruvate
Succ: succinate
YPD: yeast extract, peptone, and dextrose medium

Type 2 NADH dehydrogenases (NDH‐2) are alternative respiratory components that allow a cellular response to different environmental conditions 1. NDH‐2 have molecular masses of about 50–60 kDa, and they are typically attached to both sides of the inner mitochondrial membrane. These proteins are insensitive to rotenone but sensitive to flavone 2, 3. As NDH‐2 do not pump protons (H^+^) across the membrane, they have no direct participation in the generation of the proton electrochemical gradient 4, 5; however, NDH‐2 allow the transfer of electrons from NADH and NADPH into the respiratory chain, supporting the synthesis of ATP 6, 7.

The presence of alternative components such as NDH‐2 in the electron transport chain was first described in mitochondria of plants and fungi, and later, they were found in the plasma membrane of several Eubacteria and Archaea 8. Transcripts of these alternative elements were also present in animals from marine environments 9. The 3‐D structure of the *Saccharomyces cerevisiae* internal NADH dehydrogenase (scNdi1) corresponds to a homodimeric protein, with a hydrophobic region that allows its binding to the membrane 10, 11. A similar structure was described for *Caldalkalibacillus thermarum*'s NDH‐2 12. Besides scNdi1, *S. cerevisiae* has two additional alternative components: the external NADH dehydrogenases scNde1 and scNde2 13, 14. It has been shown that scNdi1 is part of respiratory supercomplexes 15, 16, similar to the respirasomes described in mitochondria of plant, fungi, and animal cells 17, 18, 19. An understanding of NDH‐2 is of great importance because they are potential targets of pharmacological agents against parasites of animals and plants that became resistant to traditional antibiotics 20, 21, 22. On the other hand, scNdi1 has also been proposed for gene therapy to rescue cells with defects in complex I 23, 24, 25 .

In *Ustilago maydis*, a phytopathogenic fungus of *Zea mays* which establishes a biotrophic relationship with the plant, the presence of at least one external and one internal NDH‐2 has been described, as well as an alternative oxidase (Aox) 26.

Through an *in silico* analysis, three open reading frames for NDH‐2 were found in *U. maydis* genome. The three predicted amino acid sequences (um02164, um03669, and um11333) contained two Rossman fold domains and two glycine‐rich motifs for the binding of NAD(P)H and FAD. One of these enzymes (um03669) shows a putative calcium binding domain. To evaluate the activity of NDH‐2s in *U. maydis*, oxygen consumption assays were performed in permeabilized cells cultured under glycolytic (glucose) or gluconeogenic (ethanol and lactate) growth conditions 27. The results showed the presence of rotenone‐insensitive NADH dehydrogenase activity on both sides of the inner mitochondrial membrane and an external NADPH dehydrogenase activity. Under all conditions, NDH‐2 activities were higher at the exponential phase than in the stationary phase of the growth curve. There was no activation by calcium of the alternative NADPH dehydrogenase under any condition. Transcripts of the three genes were expressed in all culture conditions, but only two of the three NDH‐2 proteins (um03669 and um02164) were found in mitochondria. Oxygen consumption rates with NADH or NADPH were different in the two growth phases of the cells.

## Materials and methods

### Materials

Analytical grade reagents were purchased from Sigma Chemical Co. (St. Louis, MO, USA), Bio‐Rad (Hercules, CA, USA), Agilent Technologies (La Jolla, CA, USA), Axygen Biosciences (Union City, CA, USA), and Millipore (Billerica, MA, USA). *Ustilago maydis* ATCC 201384 FB2 was obtained from the American Type Cell Collection (Manassas, VA, USA).

### Bioinformatic methods

To obtain the NDH‐2 sequences of *U. maydis,* a protein BLAST 28 in the *U. maydis* database (https://www.helmholtz-muenchen.de/ibis/institute/groups/fungal-microbial-genomics/resources/mumdb/index.html) was performed. Multiple sequence alignment of the NDH‐2 amino acid sequences was carried out with Clustal X 29. Pairwise identities were calculated by local sequence alignment, using SIM (http://ca.expasy.org/tools/sim-prot.html), with a gap open penalty of 12, a gap extension penalty of 4, and BLOSUM62 as the comparison matrix. MITOPROT was used for the prediction of mitochondrial signal peptides 30.

### Strains and cell cultures

*Ustilago maydis* strains were grown at 28 °C in either rich YPD medium (1.0% glucose, 0.25% peptone, and 0.5% yeast extract) or minimal medium (mm) supplemented with different carbon sources (1.0% glucose, 0.4% ethanol, or 1.0% lactate), and 0.3% ammonium sulfate as the nitrogen source, and supplemented with 1x salt solution 31. In all cases, cells were cultured in 100 mL of YPD medium for 18–24 h, harvested by centrifugation at 1000 ***g***, and washed twice with sterile H_2_O, and the final suspension was used to inoculate 1 L of medium with 4.5 × 10^8^ cells (initial OD of 0.02 at 600 nm). Cells were harvested at the exponential or stationary phases of growth and suspended in distilled H_2_O at a final ratio of 1 mL·g^−1^ wet weight. Cell growth was followed by measuring the optical density at 600 nm.

### Cell permeabilization and oxygen consumption

Plasma membrane permeabilization was achieved by incubation of *U. maydis* cells with 0.03% of digitonin for approximately 1 min inside the Clark electrode chamber, as described by Vercesi 27 and Robles‐Martínez 32. All experiments were carried out in KME buffer (100 mm KCl, 50 mm Mops/KOH, and 0.5 mm EGTA, pH 7.4). Oxygen consumption was measured in a 1.5 mL chamber at 30 °C, using a Clark‐type electrode connected to an YSI5300A biological oxygen monitor. The assays were carried out using 5 to 10 mg of cells (wet weight), and after the experiment, an aliquot of the cell suspension was used to obtain the dry weight. NADH, NADPH, pyruvate–malate, succinate, ethanol, glycerol‐3‐phosphate, and lactate were used as substrates.

### Mitochondria isolation

The method described by Sierra‐Campos 33 was used with minor modifications: Cells were collected by centrifugation at 5000 ***g*** for 5 min, 4 °C and washed twice with lysis buffer (20 mm Tris/HCl, 330 mm sucrose, 2 mm EDTA, 1 mm EGTA, 100 mm KH_2_PO_4_) and the final pellets resuspended in 1 mL of the lysis supplemented with 0.2% BSA, 0.5 mm β‐mercaptoethanol, and 1 mm PMSF. Rupture of the cells was performed at 4 °C by a mechanical method, using a Mini‐Bead Beater with glass beads of 0.5 μm of diameter. To avoid damage of mitochondria, four cycles of 30 s were selected for the rupture, with 2 min pauses on ice. The crude extract was centrifuged at 5000 ***g*** for 10 min at 4 °C. The supernatant was recovered and centrifuged at 10 000 ***g*** for 10 min at 4 °C, and the mitochondrial pellet was suspended in a small volume of lysis buffer and kept at −70 °C.

### Enzyme assays

The activities of glycolytic enzymes were determined as described by Saavedra *et al*. 34.

### Determination of protein concentration

Protein concentration was determined as described by Lowry *et al*., using bovine serum albumin (BSA) as standard.

### Sodium dodecyl sulfate polyacrylamide gel electrophoresis

Mitochondrial proteins were resolved by SDS/PAGE on 10% (w/v) polyacrylamide slab gels 35.

### Tandem mass spectrometry (LC/ESI‐MS/MS)

Mitochondrial samples were subjected to electrophoresis, and both the whole lane and a piece of the gel containing bands between 30 and 90 kDa were sent to the Proteomics Core Facility at the University of Arizona, USA, where the proteins associated with mitochondria were identified with 99.9% confidence.

### RNA extraction and RT‐PCR

To measure the RNA expression of um11333, um02164, and um03669, cells grown under the different culture conditions were harvested at the stationary phase (24 h for Glc, YPD, and EtOH, and 150 hrs for lactate), followed by RNA purification using the AxyPrep Multisource Total RNA Miniprep Kit (Axygen Biosciences). One microgram of total RNA was reverse transcribed using the protocol for gene‐specific of the iScriptTM Select cDNA Synthesis Kit. The *U. maydis* actin gene was used as endogenous control. Primers used for the cDNA synthesis and RT‐PCR are shown in Table 1.

**Table 1:** Primers used for the cDNA synthesis and the amplification of each *U. maydis* NDH‐2 transcript. After the extraction of total RNA from cells, cDNA was synthetized with the cDNA primers, following the protocol for gene‐specific described in iScriptTM Select cDNA Synthesis Kit^®^. Next, the forward and reverse primers were used to amplify short regions (200 bp) of each cDNA

Primers	cDNA	Forward DNA	Reverse DNA
um11333	5′‐AGGACGAGTCCAACAAGGAC‐3′	5′‐AGGACGAGTCCAACAAGGAC‐3′	5′‐GACCCTGGTGTGTGTACTGG‐3′
um02164	5′‐TTATCTCGCCGCACAACTAC‐3′	5′‐TTATCTCGCCGCACAACTAC‐3′	5′‐TCGCTCCTATCCTCGAAAGT‐3′
um03669	5′‐CTAGGCAGCATGTACGCACT‐3′	5′‐CTAGGCAGCATGTACGCACT‐3′	5′‐CGTCGTACTGGTCAAACACC‐3′
Actin	5′‐TTAGAAGCACTTGCGGTGCACG‐3′	5′‐GACTTGGACATCCGAAAGGA‐3	5′‐TTCGAGATCCACATCTGCTG‐3′

### Statistical analysis

To evaluate the significance of the differences between the distinct groups, a one‐way or two‐way ANOVA were applied to the data. The one‐way ANOVA was used when growth conditions (YPD, Glc‐mm, Lac‐mm, and EtOH‐mm) were tested against each respiratory substrate. The two‐way ANOVA considered two factors, growth conditions and expression of each mRNA for the PCR experiment, and growth conditions and growth phase for the activity of each respiratory substrate.

## Results and Discussion

In a previous work, some of the components of the respiratory chain of *U. maydis* mitochondria were identified using permeabilized cells 33, 36. The activity of both rotenone‐insensitive external and internal NADH dehydrogenase was described. In addition, low activities associated with both glycerol 3‐phosphate and NADPH dehydrogenases were found. Activities for lactate or alcohol dehydrogenase were not detected. Here, we analyzed the expression of some of these enzymes in cells grown under different conditions and at different stages of the growth curve.

### Bioinformatic analyses

**Figure 1:**
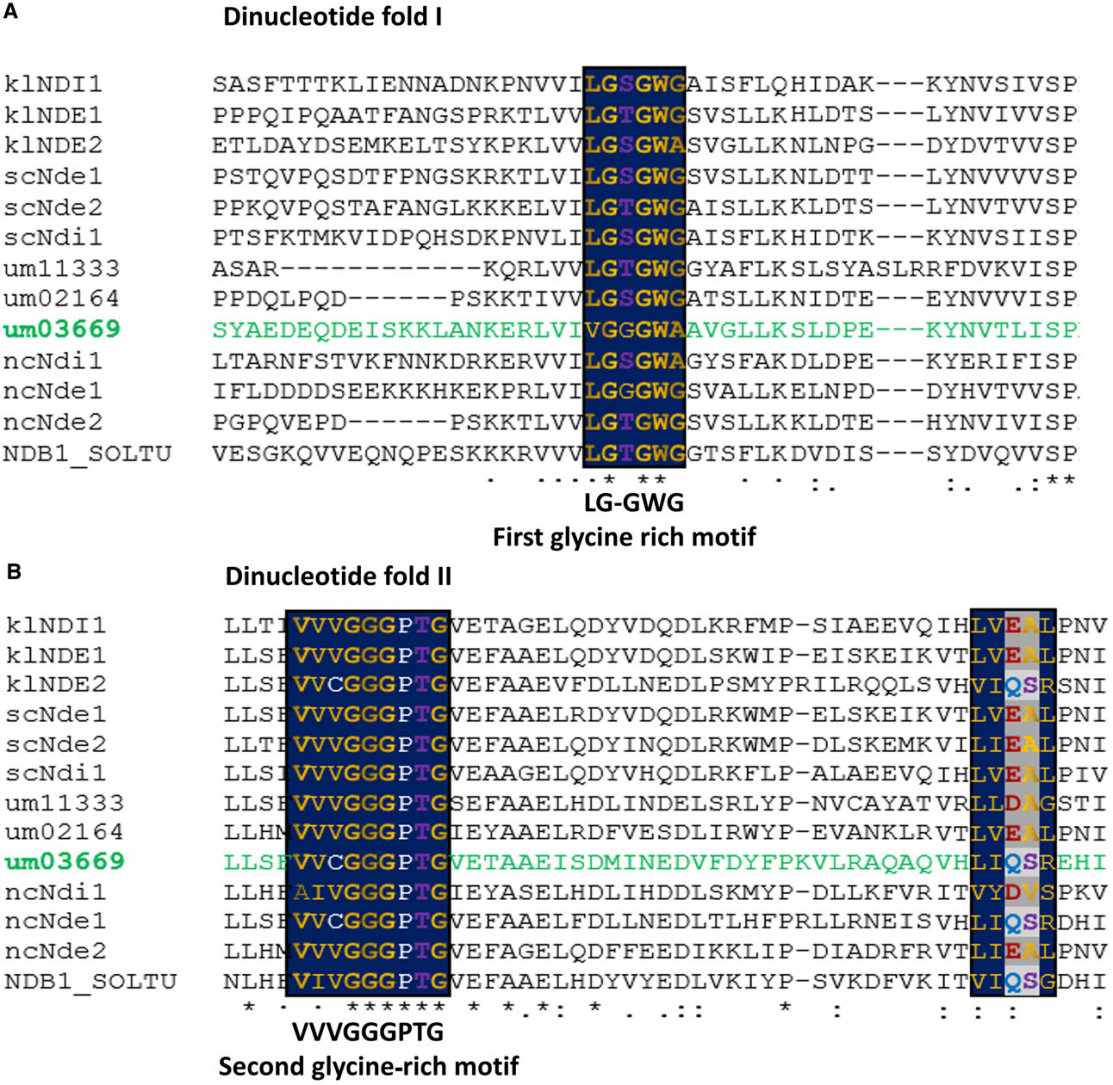
Multiple alignment using 13 NDH‐2 sequences. The first three sequences (klNDI1, klNDE1, klNDE2) correspond to *Kluyveromyces lactis* NDH‐2. The next three (scNde1, scNde2, and scNdi1) are the *NDH‐2 of Saccharomyces cerevisiae*. The following three sequences correspond to the three NDH‐2 of *U. maydis* (um11333, um2164, and um3669). The next three sequences correspond to *Neurospora crassa* NDH‐2 (ncNdi1, ncNde1, ncNde2). Finally, the last sequence corresponds to an NDH‐2 of *Solanum tuberosum*. The alignment was performed using Clustall W2 (http://www.ebi.ac.uk/Tools/msa/clustalw2). Symbols: ‘*’, the amino acid is conserved in all sequences; ‘:’, the amino acid type is conserved; ‘.’, the amino acid profile is preserved. In both panels, the glycine‐rich motifs are highlighted with blue boxes. Hydrophobic amino acids are colored yellow, residues with negative charge in red, residues with a hydroxyl group in purple, and proline and cysteine in white. Shaded in gray inside the third blue box are placed the amino acid with negative charge (D or E) or with a carboxamide group (N or Q) proposed by Desplats 37 as acidic or no acidic sequences that discriminate the activity of NADH or NADPH dehydrogenase. The um03669 sequence is colored green.

**Figure 2:**
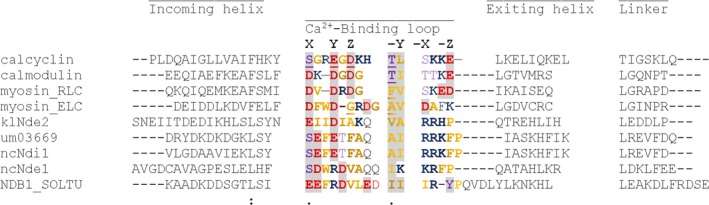
Multiple sequence alignment for the calcium binding domain (CBD). The amino acids involved in Ca^2+^‐binding are in bold type and highlighted; those that contribute with the main‐chain carbonyl oxygen are shaded in gray, as proposed by Graberek 46. Hydrophobic amino acids are colored in yellow, the residues with a positive charge in blue, with a negative charge in red, and with a hydroxyl group in purple. Calcium binding has been experimentally determined in the four mammalian proteins (calcyclin, calmodulin, myosin_RLC, and myosin_ELC); in addition, 4 fungal sequences (klNde2, um03669, ncNdi1, ncNde1) and the sequence of the plant *Solanum tuberosum* (NDB1_SOLTU) are shown.

To determine the number of NDH‐2 genes present in the *U. maydis* genome, a basic local alignment search (BLAST) was performed (https://blast.ncbi.nlm.nih.gov). The amino acid sequence of the internal NADH dehydrogenase (scNdi1) of *S. cerevisiae* was used as a query. We found three putative open reading frames for NDH‐2, *UM0216*4, *UM3669,* and *UM1133*. We found that the protein with the highest similarity to scNdi1 was um02164, with a value of 51%, and an identity of 34%; the other two amino acid sequences showed identities higher than 25% and similarities above 40%. MITOPROT (http://ihg.gsf.de/ihg/mitoprot.html) analysis of these three sequences showed the presence of mitochondrial signal peptides in each protein. The probability of exporting to mitochondria was 0.8972, 0.9896, and 0.9940 for um11333, um02164, and um03669, respectively. The alignment of 12 NDH‐2 sequences from fungi and one from plants (*Solanum tuberosum* NDB1soltu) revealed the high conservation of the two nucleotide binding motifs (Fig. 1A,B). The amino acid residues required for the binding of NADH and NADPH are highlighted in Fig. 2. In both um02164 and um11333 sequences, the classic acidic motif (EA) is present, and these enzymes might have NADH dehydrogenase activity, while um03669 has nonacidic amino acid residues (QS), suggesting that only this isoform might have both NADH and NADPH dehydrogenase activities as suggested by Desplats 37, Blaza 38, and Meng‐Shu 39.

The protein um03669 is grouped in the NDH‐2 B clade 5, 40, contains a putative calcium binding domain (CBD) (Fig. 2) with similarity to the CBD of other fungal (*Kluyveromyces lactis* and *Neurospora crassa*, klNDE2 and nceNd1), and plant (*Solanum tuberosum*, NDB1_SOLTU) NDH‐2s, where activation by calcium has been proposed 41, 42, 43, 44. However, activation by calcium was not detected in klNDE2 45. Another case in which the presence of a putative calcium binding domain was not associated with calcium activation is the NDH‐2 of *Plasmodium falciparum* (pfNDH‐2) 46. Interestingly, the um03669, as well as the other three fungal sequences analyzed in Fig. 2, lack the two essential negative residues and the hydroxylated amino acid residue required for calcium binding. These residues correspond to the *z* and *x* positions in the loop for the Ca^2+^ binding domain, defined by the X—Y‐Z—Y–X–Z motif 47.

### Cell growth in different media

To study the expression and role of the three rotenone‐resistant dehydrogenases from *U. maydis*, growth curves were obtained under the following culture conditions: YPD, glucose minimal (Glc‐mm), ethanol minimal (EtOH‐mm), and lactate minimal (Lac‐mm) media. When cells were cultured in YPD, Glc‐mm, or EtOH‐mm, the stationary phase was reached at about 24 h after inoculation (Fig. S1 and 26). As shown in Fig. S2, the concentration of glucose in the culture medium was still high after 24 h of growth. In contrast, for cells grown with other gluconeogenic carbon sources such as Lact‐mm, the stationary phase was reached at around 150 h (Fig. S1 and 26). YPD was the best condition for cell growth, with the highest biomass production (8 g wet weight·L^−1^) and a duplication time of 2.4 h. Ethanol was also a good carbon source for the generation of biomass in short times (duplication time of 3.2 h). With lactate, the duplication time was over 20 h (Fig. S1 and 26).

### Respiratory activities by permeabilized cells

**Figure 3:**
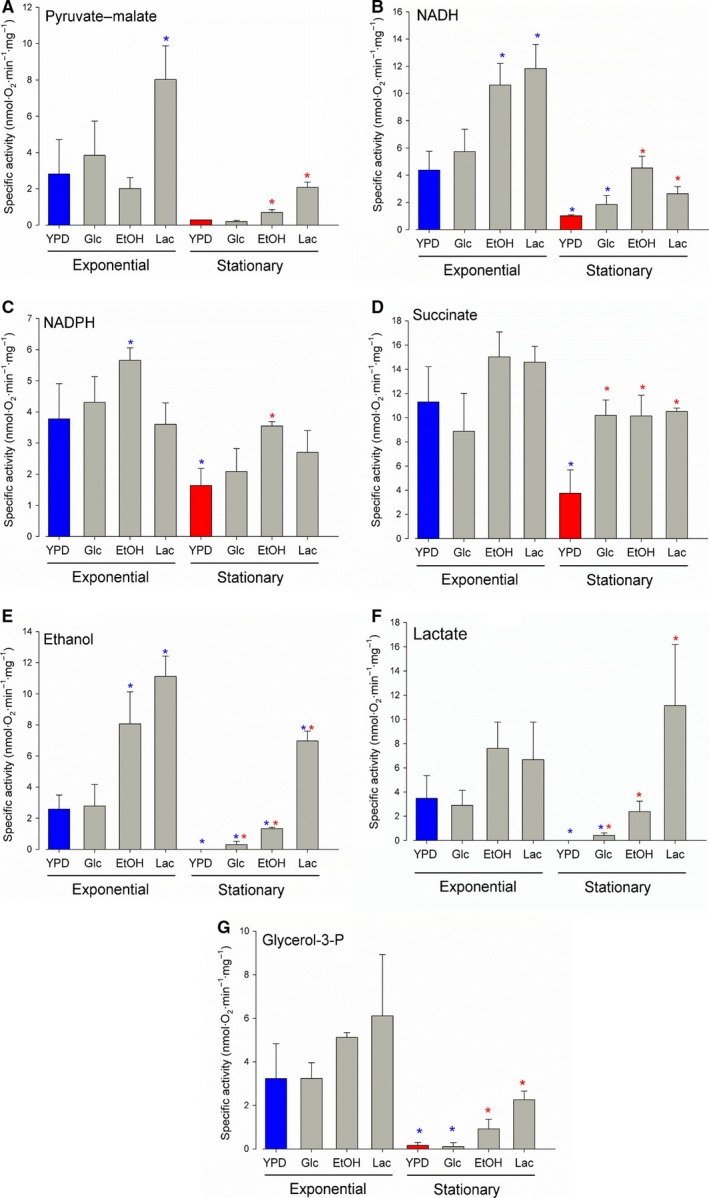
Oxygen consumption by permeabilized cells. Cells were permeabilized with digitonin inside the oxymeter chamber following standardized methods proposed by Robles‐Martínez 48. After permeabilization, different respiratory substrates were used to evaluate the rate of oxygen consumption. (A) 5 mm pyruvate‐malate, (B) 1 mm NADH, (C) 1 mm NADPH, (D) 7 mm succinate, (E) 130 mm ethanol, (F) 15 μm lactate and (G) 1 mm glycerol‐3‐phosphate. Data represent the average and standard deviation of three independent experiments. Blue asterisks on the bars indicate a significant difference with respect to the exponential‐YPD condition. Within the stationary phase, red asterisks indicate a significant difference taking as control the stationary‐YPD condition. A two‐way ANOVA was used for the analysis with a *P *< 0.05.

**Figure 4:**
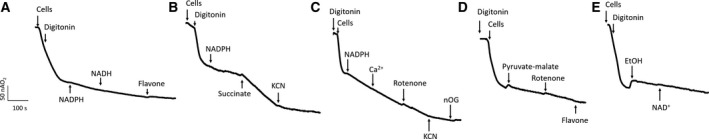
Time courses of oxygen consumption by permeabilized *U. maydis* cells harvested at the exponential phase. Cells were permeabilized with digitonin inside the oxymeter chamber following standardized methods proposed by Robles‐Martínez 48. After permeabilization, oxygen consumption was stimulated by the addition of the indicated substrates. (A) NADPH, NADH and flavone. (B) NADPH, succinate, and KCN. (C) NADPH plus calcium, followed by rotenone, KCN and nOG. (D) To discriminate between complex I and the internal NDH‐2 activities, pyruvate‐malate was used as substrate, and rotenone plus flavone to inhibit the respiratory chain activity. (E) Ethanol plus NAD ^+^. For permeabilization of cells, digitonin at 0.03% (v/v) was used. The concentration of substrates was: 10 mm succinate, 130 mm ethanol, 1 mm NADH, 1 mm NADPH, and 5 mm of both pyruvate and malate. Inhibitors were used at the following concentrations: 10 μm rotenone, 47 μm flavone, 1 mm potassium cyanide (KCN), 1 μm *n*‐octylgalate (nOG). Calcium was used at a concentration of 1.2 mm, and NAD^+^ at 1 mm.

Next, cells harvested at the exponential or stationary phases were permeabilized in the same Clark electrode chamber with digitonin at a final concentration of 0.03–0.2% (w/v), as described by Vercesi 27. Oxygen consumption assays in permeabilized cells were performed with different substrates and inhibitors (Figs 3 and 4). Both NADH and NADPH were used to assess the participation of the alternative external NADH and NADPH dehydrogenases. Pyruvate–malate was used to evaluate the activity of the internal NADH dehydrogenase. In all cases in which the NDH‐2 was studied, the experiments were carried out in the presence of 10 μm of rotenone. The concentration of rotenone used in the assay was sufficient to inhibit complex I but not the NDH‐2 36. To evaluate other electron input sites in the respiratory chain of *U. maydis* mitochondria, ethanol, succinate, glycerol 3‐phosphate, or lactate was used to assess the participation of each specific dehydrogenase in both growth phases. As can be seen in Figs 3 and 4, permeabilized cells showed external rotenone‐resistant NADH and NADPH dehydrogenase activities in both the exponential and stationary phases, regardless the culture conditions. The internal NADH dehydrogenase activity was found in both growth phases with ethanol and lactate as carbon sources, but with glucose, this activity was mostly present in the exponential phase.

In general, activities were higher in the exponential phase than in the stationary phase for all the culture conditions (Fig. 3). Although oxygen consumption was higher with NADH than with NADPH, both activities were within the same order of magnitude. All the pathways feeding the electron transport chain were active in the exponential phase, independently of the culture media. On the other hand, in the stationary phase there was a large decrease in oxygen consumption driven by pyruvate–malate, NADH, glycerol 3‐phosphate, lactate, and ethanol. This was more evident in cells obtained from YPD and Glc‐mm, in which some of the activities were undetectable. It is worth noting that oxygen consumption rates with NADH and ethanol as respiratory substrates were twofold higher in cells grown in nonglycolytic carbon sources (EtOH‐mm and Lac‐mm) in comparison with media containing glucose, and this observation occurred in both, the exponential and stationary phases. Ethanol was a good respiratory substrate, mostly in cells from the exponential phase of growth, and oxygen consumption was not altered by the addition of NAD^+^ (Figs 3 and 4E), suggesting that the alcohol dehydrogenase is located in the mitochondrial matrix. In contrast to lactate, ethanol supported a rapid growth of the cells, resembling the growth kinetics obtained in the presence of glucose. This finding is interesting because there is no lactic or alcoholic fermentation in *U. maydis* yeast 34, 36, but cells can consume very efficiently ethanol as a carbon source and as a respiratory substrate. The results also show that the expression of the alcohol, glycerol 3‐phosphate, and lactate dehydrogenases was not specific for each culture condition. Thus, when cells were grown in ethanol, respiratory activities in permeabilized cells can be supported by lactate and glycerol 3‐phosphate. It seems that when *U. maydis* yeasts are cultured in media with gluconeogenic carbon sources (ethanol and lactate) or under conditions of high energy demand (exponential phase), several pathways that feed the electron transport chain are expressed in mitochondria. However, we found that during the exponential phase, the activity of some components (external NADH‐DH, G3P‐DH, lactate‐DH, and EtOH‐DH) was lower in cells cultured in the presence of glucose (Fig. 3). This decrease was more evident during the stationary phase, in which the activities of the G3P‐DH, lactate‐DH, and EtOH‐DH were insignificant in cells cultured in the presence of glucose (Fig. 3). It should be noted that the concentration of glucose in the culture medium is still high in the stationary phase (Fig. S2) 48.

Next, we studied the activity of the respiratory chain of *U. maydis* to sequential additions of different substrates. Regardless of the order of addition of saturating concentrations of either NADH or NADPH, there was no increase in the oxygen consumption upon the addition of the second substrate, for example, adding NADH after NADPH did not increase the respiratory activity (Fig. 4A). This result suggests that both substrates interact with the same NDH‐2 enzymes. In contrast, once the saturation with NADH or NADPH was reached, a subsequent addition of succinate, pyruvate–malate, or glycerol 3‐phosphate increased the rate of oxygen consumption (Fig. 4B). Figure 4C shows that NADPH dehydrogenase activity did not respond to high concentrations of calcium, as occurs in other fungal systems such as *N. crassa* and some plants 41, 44. As both the mRNA and the protein with the calcium binding domain (um03669) were synthesized in the four culture conditions, the lack of an effect of calcium on the respiratory activity of permeabilized cells is in agreement with the absence of critical residues in the putative calcium binding domain of this protein. The experiment displayed in Fig. 4D suggests the presence of an internal NDH‐2, insensitive to rotenone but sensitive to flavone.

### RNA and protein expression

**Figure 5:**
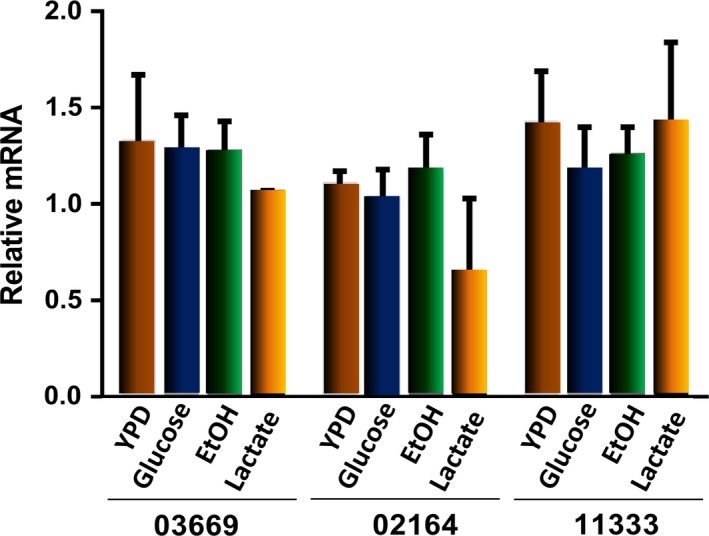
Reverse transcription‐polymerase chain reaction of the NDH‐2s transcripts in *Ustilago maydis*. Cells grown under different conditions and harvested at the exponential phase were disrupted to obtain total RNA. Using the primers described in Table 1, RT‐PCR was carried out to evaluate the levels of each transcript using the actin transcript for normalization. Data represent the average of three independent experiments.

Expression of the three *U. maydis* NADH dehydrogenases was analyzed by RT‐PCR in cells harvested at the stationary phase of growth. For normalization, the actin mRNA was used as reference 49. Figure 5 shows similar levels of expression for the three RNA transcripts in cells cultured under different conditions. Proteomic analysis further revealed that only the NADH dehydrogenases um02164 and um03669 were associated with mitochondria of *U. maydis* cells cultured in EtOH‐mm, Lac‐mm, and YPD culture media and harvested at the exponential phase of growth (Table 2), while peptides of um11333 were not detected in mitochondria. Taken together, these results suggest that in *U. maydis* a posttranscriptional regulation may exist that allows the differential translation of certain mRNA. In addition, due to the important role of these enzymes in the intermediary metabolism, regulation by covalent modifications like phosphorylation–dephosphorylation should be considered. In this sense, there are several putative phosphorylation sites in the amino acid sequences of the three NDH‐2 from *U. maydis* (data not shown). In support of this suggestion, phosphorylation of scNDI1 and scNDE1 has been reported 50, 51.

**Table 2:** Representative enzymes of some metabolic pathways found in cells cultured under different culture conditions. The samples were sent to the research laboratory in Arizona, USA, as described under the materials and methods. The numbers represent a weighted value of the abundance of each protein under each condition. 6‐P‐gluconate dehydrogenase, 6 phosphogluconate dehydrogenase; Aox, alternative oxidase; FBPase‐1, fructose bisphosphatase 1; G3PDH, glyceraldehyde 3 phosphate dehydrogenase; Glc‐6‐P dehydrogenase, glucose‐6‐phosphate dehydrogenase; Glycerol‐3‐P dehydrogenase, glycerol‐3‐phosphate dehydrogenase; GPI, glucose 6 phosphate isomerase; ND, no determined; ND2, alternative NADH dehydrogenase 2; PEP‐CK, phosphoenolpyruvate carboxykinase; PGK, phosphoglycerate kinase; PK, pyruvate kinase; TPI, triose phosphate isomerase

Enzyme	Accession number	YPD	EtOH‐mm	Lac‐mm
Glycolysis				
GPI	A0A0D1DUK2	5	12	5
Aldolase	A0A0D1E8V6	6	12	12
TPI	A0A0D1DYK6	ND	9	6
G3P dehydrogenase	A0A0D1CU24	7	13	19
PGK	A0A0D1DSH4	12	13	12
Enolase	A0A0D1E2E4	11	13	9
PK	A0A0D1E735	14	22	15
Gluconeogenesis				
FBPase‐1	A0A0D1C717	ND	8	ND
PEP‐CK	A0A0D1E2J3	ND	32	25
Phosphate pentose pathway				
Glc‐6‐P dehydrogenase	A0A0D1BZD5	5	4	ND
6‐P‐gluconate dehydrogenase	A0A0D1CRJ9	13	12	17
Transaldolase	A0A0D1DYQ2	3	5	6
Transketolase	A0A0D1CJR1	8	10	14
Krebs cycle				
Citrate synthase	A0A0D1CAY8	23	20	18
Aconitase	A0A0D1DXN8	28	26	38
Isocitrate dehydrogenase	A0A0D1E633	17	18	15
2‐oxoglutarate dehydrogenase	A0A0D1CAI6	15	7	11
Succinyl‐CoA synthase	A0A0D1E476	12	9	9
Succinate dehydrogenase	A0A0D1DVG5	16	41	43
Fumarase	A0A0D1E945	12	6	11
Malate dehydrogenase	A0A0D1BZ90	6	20	18
Respiratory complexes and ATP synthase				
Complex I	A0A0D1DQS4	15	26	33
Complex II	A0A0D1CDW6	16	41	43
Complex III	A0A0D1CAD4	24	28	26
Complex IV	A0A0D1DRD6	13	14	11
Complex V	A0A0D1E227	45	62	57
Respiratory alternative elements				
Lactate dehydrogenase	A0A0D1E9U3	ND	7	27
ND2 um02164	A0A0D1E082	7	18	24
ND2 um03669	A0A0D1DV23	24	7	16
Glycerol‐3P dehydrogenase	A0A0D1CVJ0	15	10	21
Aox	A0A0D1C7C6	ND	11	3

### The role of the alternative NAD(P)H dehydrogenases in *Ustilago maydis*

**Figure 6:**
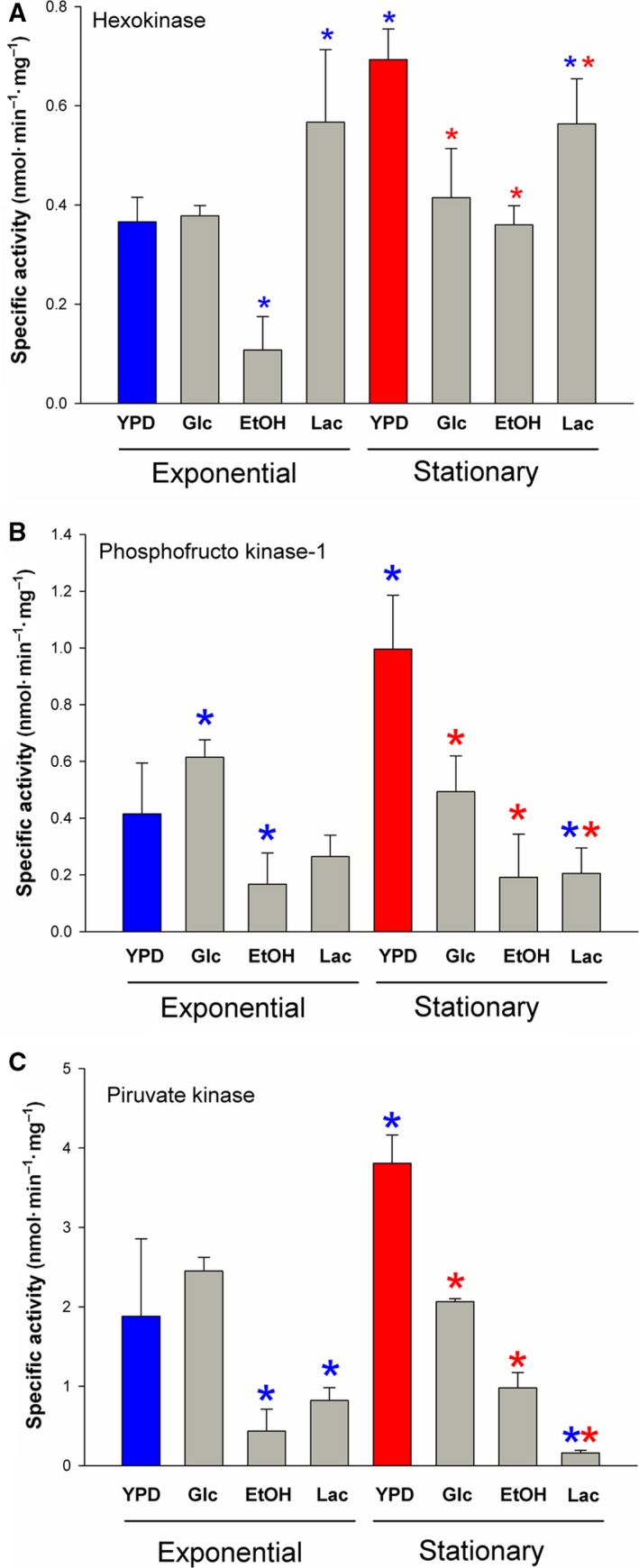
Specific activities of hexokinase, phosphofructokinase‐1, and pyruvate kinase in *U. maydis* cell extracts. Enzyme activity was determined as described in Saavedra *et al*. 34. Data represent the average and standard deviation of five independent experiments. (A) For hexokinase, the activity was measured in a reaction mixture containing 5 mm MgCl_2_, 1 mm NADPH, 5 mm glucose, 2 U G6PDH, 13–68 mg of cytosolic extract. The reaction was started by the addition of 3 mm ATP. (B) For the phosphofructokinase‐1, the reaction mixture contained 5 mm MgCl_2_, 1 mm EDTA, 0.15 mm NADH, 3 mm ATP, 0.5–1 U aldolase, 3 U Gly3PDH – 4.5 U triose phosphate isomerase (Roche) and 30–400 mg of cytosolic extract. The reaction was started by the addition of 2.5 mm fructose 6 phosphate. (C) Pyruvate kinase was assayed in a reaction mixture containing 10 mm MgCl_2_, 0.15 mm NADH, 1 mm phosphoenopyruvate, 2.5 U lactate dehydrogenase, 13–90 mg of cytosolic extract. The reaction was started with 3 mm ADP. Blue asterisks on the bars indicate a significant difference with respect to the exponential‐YPD condition. Within the stationary phase, red asterisks indicate a significant difference taking as control the stationary‐YPD condition. A two‐way ANOVA was used for the analysis with a *P *< 0.05.

To place these dehydrogenases in the metabolic context of the cells, information about the presence of some proteins belonging to central metabolic pathways under the three culture conditions (YPD, EtOH‐mm, and Lac‐mm) was obtained through proteomic analysis. In general, the expression of the enzymes that belong to carbon metabolism was not associated with the culture conditions of the cells. For example, with the gluconeogenic substrates ethanol and lactate, the three classic regulatory glycolytic enzymes, hexokinase, phosphofructokinase‐1, and pyruvate kinase were expressed (Fig. 6). During the exponential phase, there was no difference between the YPD and Glc‐mm in the activities of the three enzymes, but with ethanol as the carbon source, there was a large decrease in their activities. Similarly, in EtOH‐mm and Lac‐mm, there was a decrease in the activities of PFK‐1 and PK. When cells entered the stationary phase, the activities of the three enzymes in YPD increased twice, but this was not observed in Glc‐mm. Regarding the PFK‐1 and PK, their activities were lower in EtOH‐mm and Lac‐mm than in YPD or even Glc‐mm. Interestingly, the activity of the hexokinase is high in all conditions, with the exception of ethanol in the exponential phase. In short, it can be said that with the exception of the HK, the activities of the two glycolytic enzymes were smaller in cell growing with the two gluconeogenic substrates, ethanol and lactate. Given the relative high activity of regulatory glycolytic enzymes in cells growing in gluconeogenic media (Fig. 6), it is expected that some type of control mechanism occurs to avoid energetically expensive futile cycles. Table 2 shows that enzymes from glycolysis, Krebs cycle, pentose phosphate pathway, and gluconeogenesis were present in cells regardless of the carbon source (glucose, ethanol, or lactate). The mitochondrial flavoenzyme lactate dehydrogenase was expressed mainly in the presence of lactate, and to a lesser extent with ethanol, but not in YPD. In contrast, the mitochondrial glycerol 3‐phosphate dehydrogenase and the two alternative NADH dehydrogenases were expressed under all the conditions.

Activity of the mitochondrial external NADH dehydrogenase makes sense in the context of *U. maydis* metabolism. As this organism is fully respiratory, these enzymes are essential for the continuous operation of glycolysis and other metabolic routes that depend on the availability of NAD^+^. In contrast, the role of the NADPH dehydrogenase activity is more difficult to explain, because in many anabolic pathways, NADPH is used as substrate. In a previous work, we found that the activities of the enzymes in the oxidative phase of the phosphate pentose pathway 52 are more active than the glycolytic enzymes reported by Saavedra *et al*. 34. Thus, it is possible that the NADPH dehydrogenase activity in mitochondria allows the rapid and efficient oxidation of NADPH produced in the pentose pathway.

*Ustilago maydis* has been extensively used for years as a model to study the participation of several signal transduction pathways in the yeast‐mycelium transition and pathogenicity 53, 54, or more recently the introduction of this microorganism for biotechnological applications 55, 56. In sharp contrast with this vast information, the mechanisms underlying the metabolic changes described in this work are still unknown. Cloning and expression of the three NADH dehydrogenases will allow the determination of their specificities and kinetic parameters, and the evaluation of the effect of calcium on the activity of the um03669 enzyme.

## Author contributions

AP conceptualized and coordinated the research project, provided funding to support the research, and reviewed and edited the manuscript. JPP conceptualized and coordinated the research project, analyzed the data, provided funding to support the research, and reviewed and edited the manuscript. GGS conceptualized the research project, provided funding to support the research, and reviewed the manuscript. GMO conceptualized, coordinated, and developed the research project; performed the bioinformatics analyses, PCR and protein data analyses, and oxygen consumption assays; and wrote the original draft. DMM isolated mitochondria, performed SDS/PAGE with mitochondrial samples, analyzed the data, and reviewed the manuscript. CCM performed PCR analyses and oxygen consumption assays analysis. JCV determined the glycolytic enzyme‐specific activities and analyzed the data. LRA performed yeast growth and glucose determination, and performed SDS/PAGE with mitochondrial samples. HVM isolated mitochondria, protein analysis, and oxygen consumption assays.

## Supporting information


**Fig. S1**. *Ustilago maydis* growth curves in different culture media. Panel A shows the growth of *U. maydis* when the carbon source was glucose or ethanol. Panel B shows the growth in media with lactate as the carbon source. Tables show the growth rate constants (*k*) and duplication times (g).

**Fig. S2**. *Ustilago maydis* growth in YPD medium. Yeast growth was followed by absorbance at 600 nm. Simultaneously, the residual concentration of glucose in the culture medium was determined using a kit based on the glucose oxidase activity (Spinreact ®). Standard deviations for each point were obtained from three independent experiments.
