# Supplementary figures and images for: Expression of alternative NADH dehydrogenases (NDH‐2) in the phytopathogenic fungus Ustilago maydis
Source: FEBS Open Bio. 2018 Jul 5;8(8):1267–79. doi: 10.1002/2211-5463.12475 (PMC6134880; doi:10.1002/2211-5463.12475)

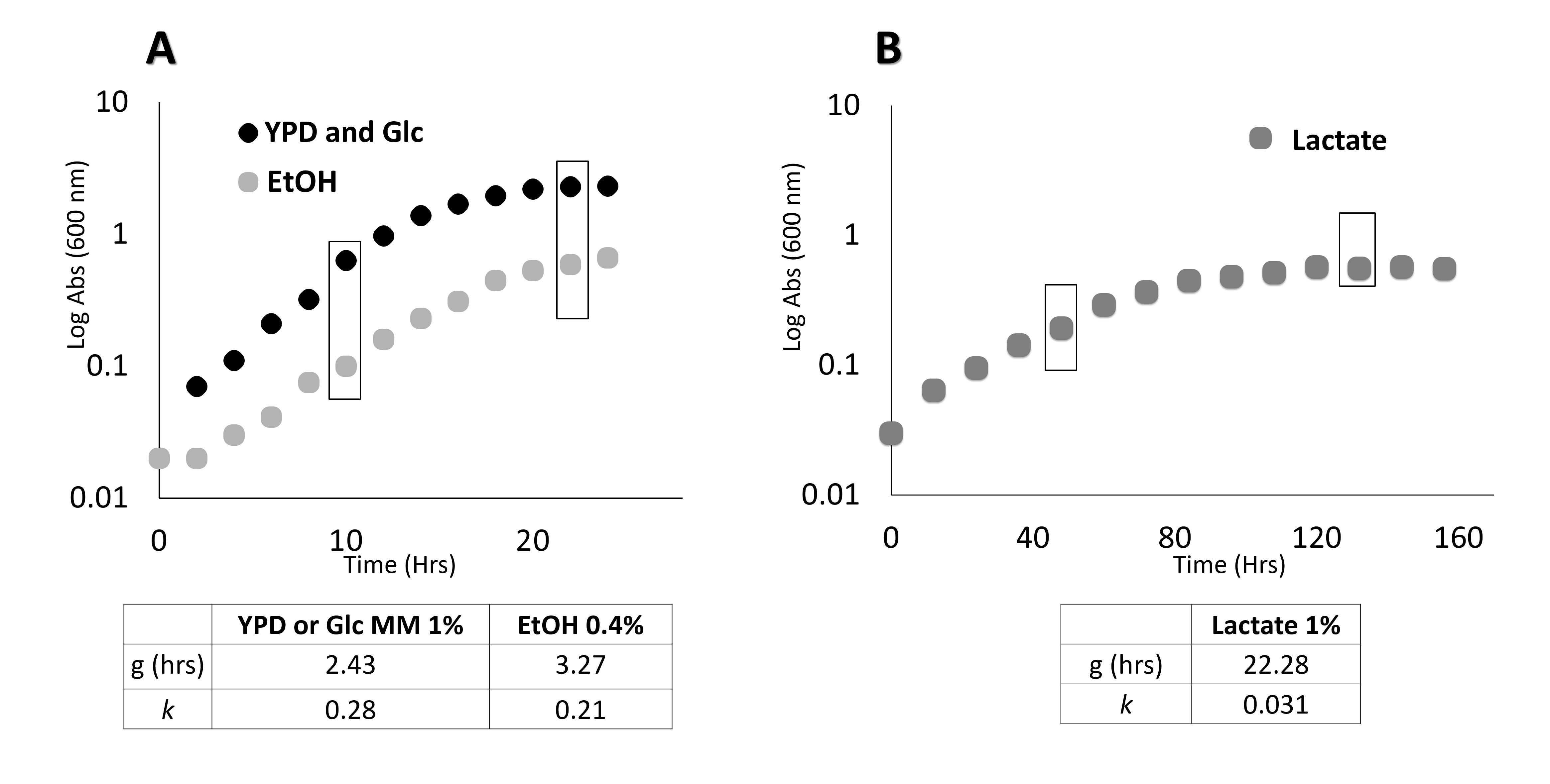

Supplement: Supplementary file 1 — Fig. S1. Ustilago maydis growth curves in different culture media. Panel A shows the growth of U. maydis when the carbon source was glucose or ethanol. Panel B shows the growth in media with lactate as the carbon source. Tables show the growth rate constants (k) and duplication times (g). [file FEB4-8-1267-s001.tif]

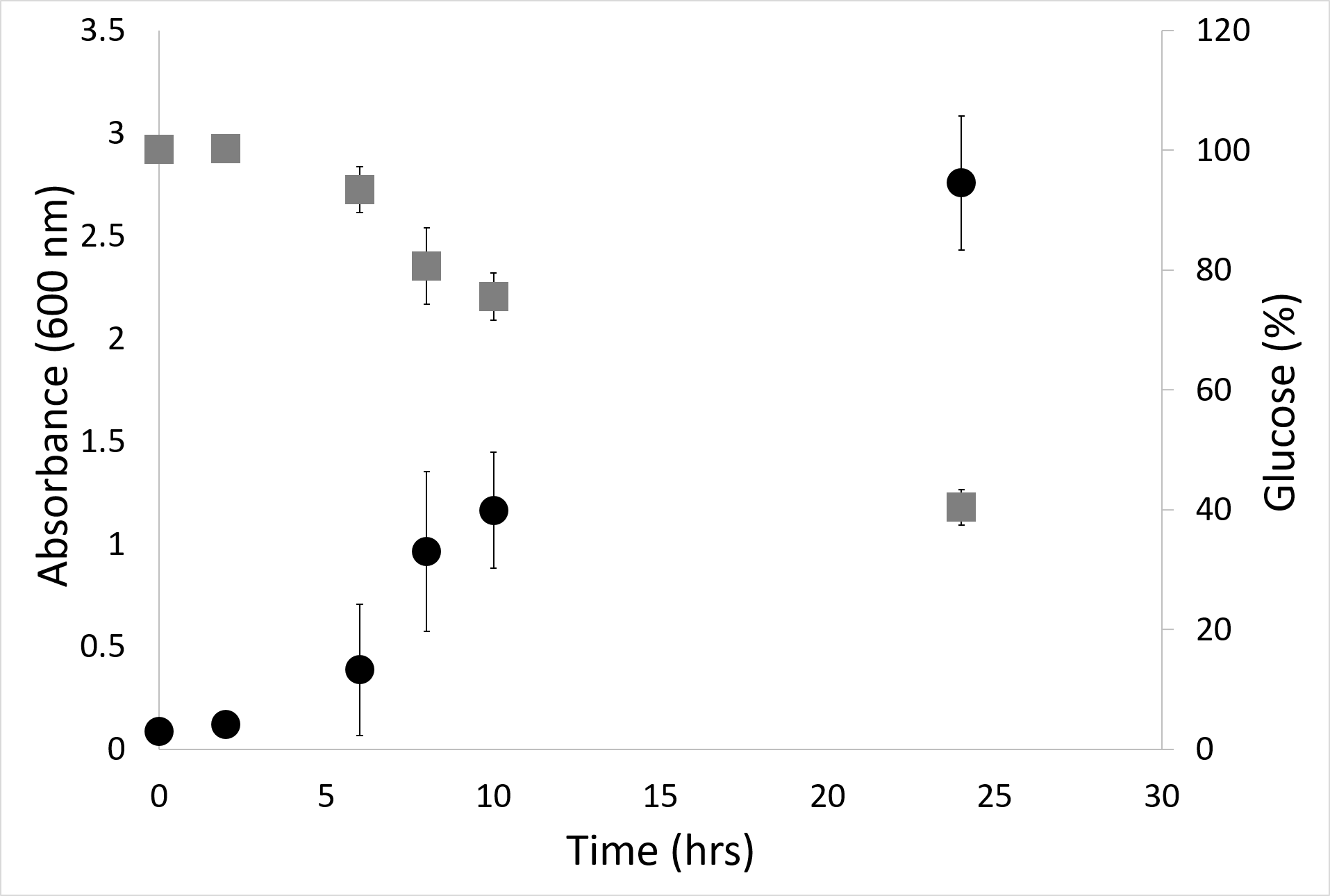

Supplement: Supplementary file 2 — Fig. S2. Ustilago maydis growth in YPD medium. Yeast growth was followed by absorbance at 600 nm. Simultaneously, the residual concentration of glucose in the culture medium was determined using a kit based on the glucose oxidase activity (Spinreact ®). Standard deviations for each point were obtained from three independent experiments. [file FEB4-8-1267-s002.tif]
